# Social network analysis of rural medical networks after medical school immersion in a rural clinical school

**DOI:** 10.1186/s12913-019-4132-z

**Published:** 2019-05-14

**Authors:** Denese E. Playford, Tessa Burkitt, David Atkinson

**Affiliations:** 1 The Rural Clinical School of Western Australia, RCSWA, M706, The School of Medicine, The University of Western Australia, Crawley, WA 6009 Australia; 2 The University of Notre Dame Australia, Fremantle Campus, Western Australia, Australia, 32 Mouat St, Fremantle, WA 6160 Australia

**Keywords:** Rural clinical school, Rural, Medical, Workforce, Social network analysis, Recruitment

## Abstract

**Background:**

The impact of new medical graduates on the social dimensions of the rural medical workforce is yet to be examined. Social Network Analysis (SNA) is able to visualize and measure these dimensions. We apply this method to examine the workforce characteristics of graduates from a representative Australian Rural Clinical School.

**Methods:**

Participants were medical graduates of the Rural Clinical School of Western Australia (RCSWA) from the 2001–2014 cohorts, identified as being in rural work in 2017 by the Australian Health Practitioner Regulation Agency. SNA was used to examine the relationships between site of origin and of work destination. Data were entered into UCInet 6 as tied pairs, and visualized using Netdraw. UCINet statistics relating to node centrality were obtained from the node editor.

**Results:**

SNA measures showed that the 124 of 709 graduates in rural practice were distributed around Australia, and that their practice was strongly focused on the North, with a clear centre in the remote Western Australian town of Broome. Women were strongly recruited, and were widely distributed.

**Conclusions:**

RCSWA appears to be a “weak tie” according to SNA theory: the School attracts graduates to rural nodes where they had only passing prior contact. The multiple activities that comprise the social capital of the most attractive, remote, node demonstrate the clear workforce effects of being a “bridge, broker and boundary spanner” in SNA terms, and add new understanding about recruiting to the rural workforce.

## Background

It is known that attracting new medical graduates to rural settings is difficult in all parts of the world, resulting in striking differences in distribution between the urban and the rural medical workforce, for example, in South Africa [1], Canada [2], Australia [3], America [4], Thailand [5] and throughout the developing world [6].

However, a rising tide of data shows that the Australian experiment with “Rural Clinical Schools” (RCSs), which place pre-graduate medical students in a rural clerkship for at least one academic year [7], is having flow on workforce effects. Not only are there disproportionate number of RCS graduates entering rural work in general [8–10], but these graduates are moving further and living more remotely than non-RCS grads [11, 12]. They may also be staying longer than would be expected of new graduates [13, 14].

The impact of these new graduates on the social dimensions of the rural medical workforce has not been examined. However it is reasonable to suppose that an influx of young, newly qualified doctors will have an impact on the social capital of the rural towns to which they relocate [15]. It is also possible that this “RCS workforce phenomenon” may create a new, positive culture that is conducive to ongoing medical recruitment. For example carefully controlled projects, such as the Framingham Heart Study, have shown that positivity can, and does, diffuse through a network [16]. We therefore propose that the geographical re-distribution of RCS alumni is establishing new workforce networks in rural Australia. We examine this hypothesis using Social Network Analysis (SNA), which is designed to describe relationships in a way that advances their analysis [17].

The assumption of SNA is that all individuals are embedded in a series of relationships which either constrain or enable social behavior [18]. SNA is able to both display and analyse the relationships between groups and individuals. By visualizing and quantifying patterns within networks, SNA is able to depict social interactions and to measure their effect [19] . These characteristics make SNA the ideal methodology for assessing whether there are any patterns in RCS graduates’ workplace choices. To date no such analysis has been carried out. However, these data are important in further understanding the RCS phenomenon - which is a prototype of educational interventions intended to have workforce effect - with respect to workforce distribution to even the most remote parts of a country, through identifying the extent to which social networks may be involved in workforce development, as opposed to more conventionally examined factors such as financial benefits, and geographical attractiveness [20].

## Methods

**Fig. 1:**
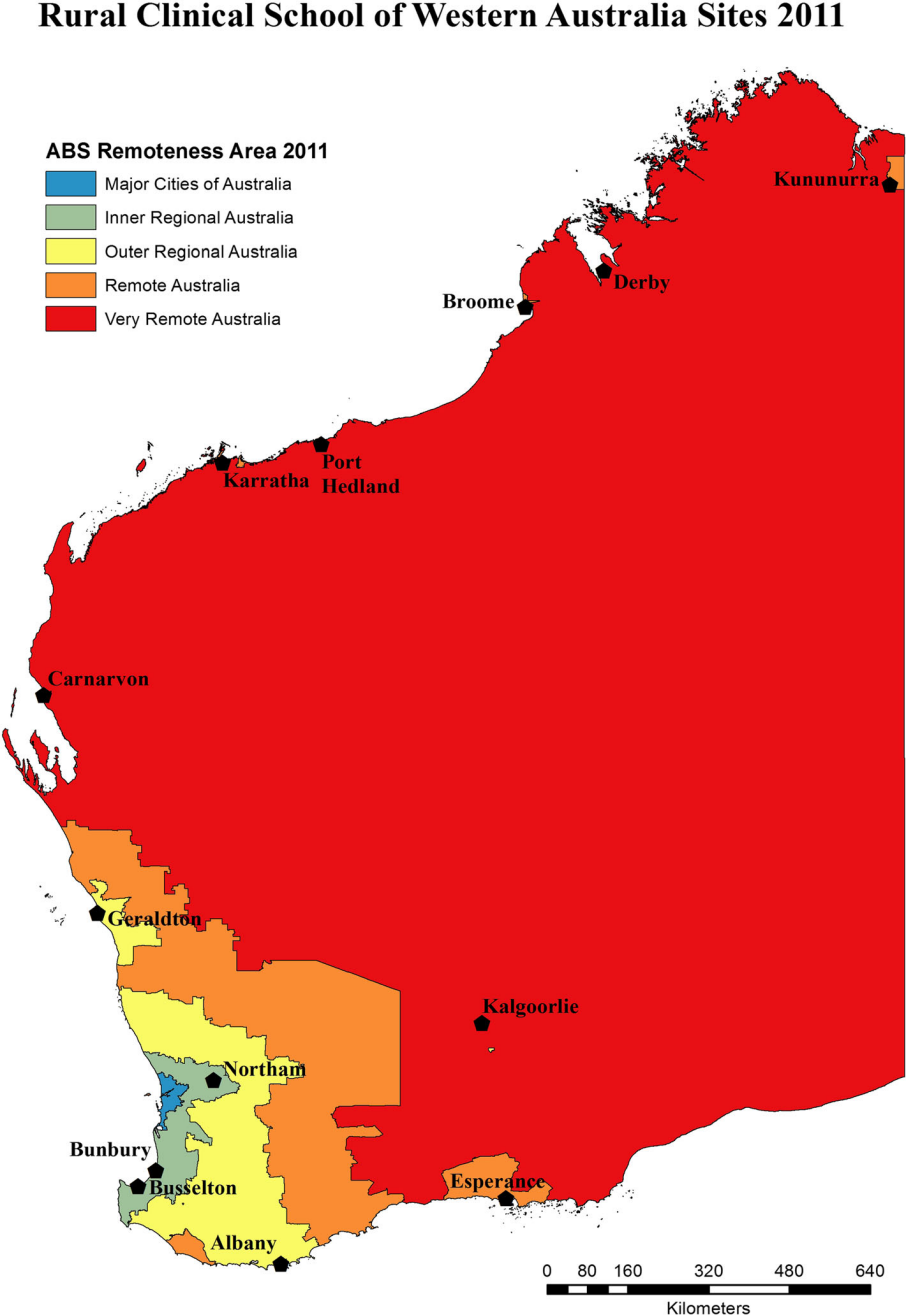
Map of Western Australia showing the location of RCSWA undergraduate placement sites. Note the most remote site, Kununurra, is 4000 km north of the capital city, Perth (dark black area). The Figure has been constructed from an existing publicly available map sourced from Australian Bureau of Statistics (ABS) Standard Geographical Classification - Remoteness Areas, 2011, obtained from 1216.0.15.001 - Australian Standard Geographical Classification (ASGC) - Electronic Structures, July 2011

The participants in this study were medical graduates of the Rural Clinical School of Western Australia (RCSWA). RCSWA is a longitudinal integrated clerkship which competitively selects 25% of the penultimate clinical year from both medical programmes in Western Australia to live and work in a rural or remote town within Western Australia for one academic year. Students with and without prior interest in rural practice are selected [21]. The distribution of sites in which these students are placed is shown in Fig. 1, which depicts sites in all remoteness classifications from inner regional (eg Bunbury) to remote (eg Kununurra). In their first postgraduate year (PGY1), graduates have very restricted placement choices in Western Australia, however by post graduate year two (PGY 2), they are able to begin making career-related choices, so graduates were included from their second postgraduate year.

The inclusion criterion (boundary specification) was defined as including all RCSWA graduates from the 2001–2014 cohorts who were identified as being in rural work in the 2017 data collection. Data were collected from the publicly available Australian Health Practitioner Regulation Agency (AHPRA), which lists the individual’s primary practice location every year. Rural work was defined according to the Australian Standard Geographical Classification – Remoteness Area [22] as RA2–5, where RA2 is inner regional, and RA5 is very remote. Graduates entering urban work (RA1, major city) were not included. Previous work showed that for durations of one year of placement, AHPRA locations were 88% concordant with direct personal communication [23] and so AHPRA data was assumed to be a reliable source of location information.

Data were entered into UCInet 6 as tied pairs: RCSWA site of origin for medical students’ placement was specified as the “from” node, and rural town of destination as graduates was specified as the “to” node. Each graduate was represented by one “edge” or line between the “from” and “to” points, so that all the lines represent a single doctor, with the exception of the line strength figure which shows multiple doctors’ connections to the same destination. All the connections were unidirectional, that is the lines were directed from site of origin for the doctor’s RCSWA year, to the site of rural work in 2017.

As a way of further defining the attributes of the destination town, other qualities such as the state (WA vs not WA), and the gender most frequently attracted to that town were added.

We examined the whole network of single connections for all rural-working graduates, since we were interested in whole-of-school outcomes with respect to return from a rural training site to a rural work site. These data collectively constituted a “directional graph” in which relationships are depicted.

Statistics relating to the relationships that graduates had with different towns were obtained from the node editor in the UCINet programme. The statistic relating to the number of direct connections with a town was assessed through the “degree” measure for that town. The statistic relating to the position of the destination town in relation to other towns, for example how many placement towns contributed graduates to that workforce destination, were assessed through the “between-ness” measure. The statistic relating to the shortest connection (“path”) between the workforce town and all other towns were measured through the “closeness” measure [24].

Each destination node represented a rural town to which attribute data, such as State (Western Australia vs not Western Australia), could be added. For illustrative purposes, the gender involved in the tie was added as an attribute of the town, making it either more male- or more female- oriented, given the gender of the graduate/s who moved there. Where there were multiple doctors migrating to the town, the most frequently attracted gender was attributed to the town.

Reflexive ties, where medical students were trained in one town and then returned to the same town as doctors, could not be charted in Netdraw, and so were tabulated.

Ethics was obtained from the University of Western Australia Human Research Ethics Committee RA/4/1/1627. All participants gave their consent in writing.

## Results

**Fig. 2:**
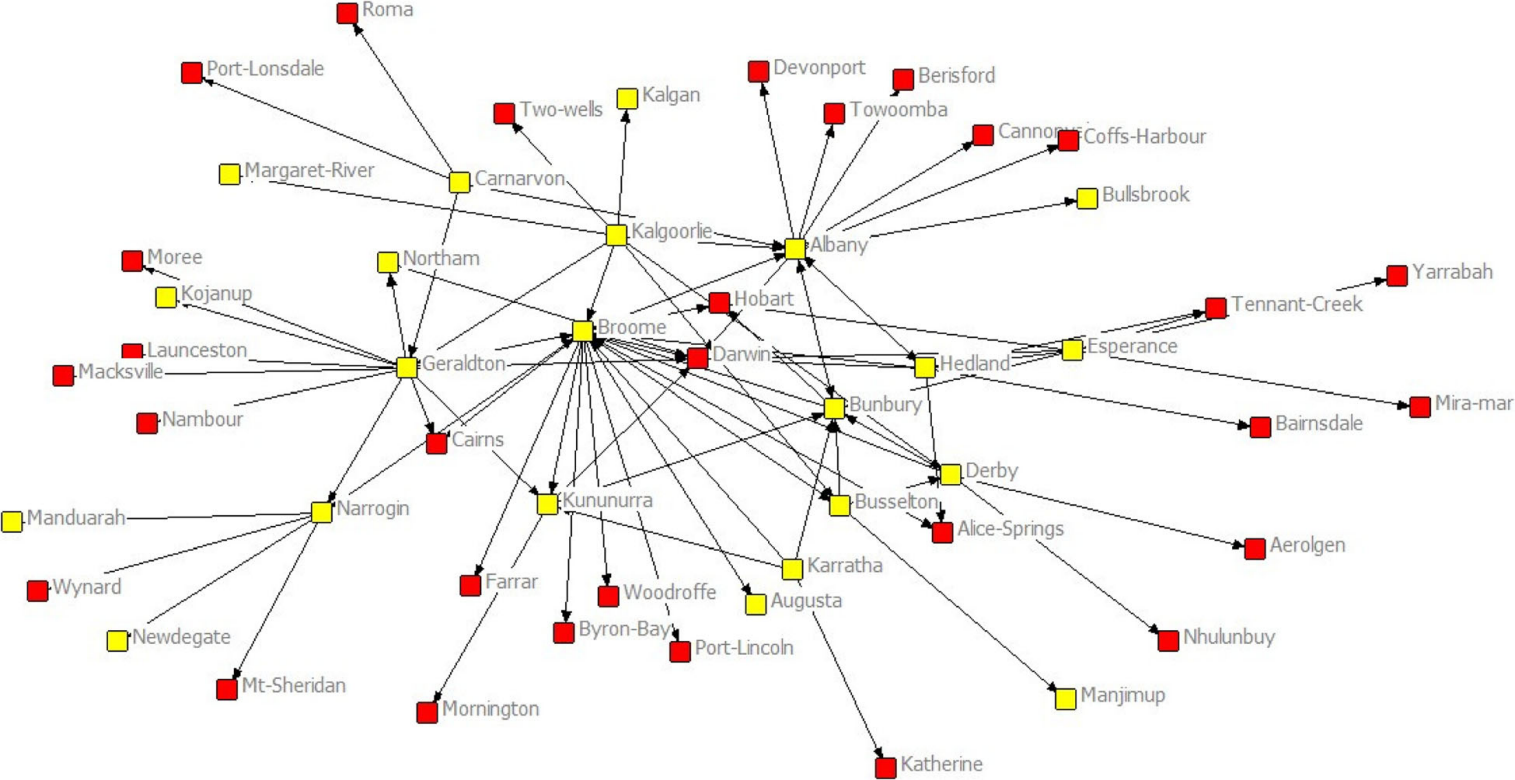
Social Network of Rural Clinical School of WA Edge Array showing Western Australia -remaining graduates (Yellow icon) versus location in other states in Australia (Red icon)

Of 709 PGY 2 – PGY 15 graduates in 2017, 17.5% (124) were in rural practice as identified by principal location in AHPRA. Nearly two thirds (75/124) of these graduates from 2002 to 2013 RCSWA cohorts were located in rural Western Australia in 2017 – that is they remained in the same state as their RCS site. Figure 2, where each node’s attribute was given as either “Western Australia” or “not Western Australia” shows that most ties were to Western Australian towns.

**Fig. 3:**
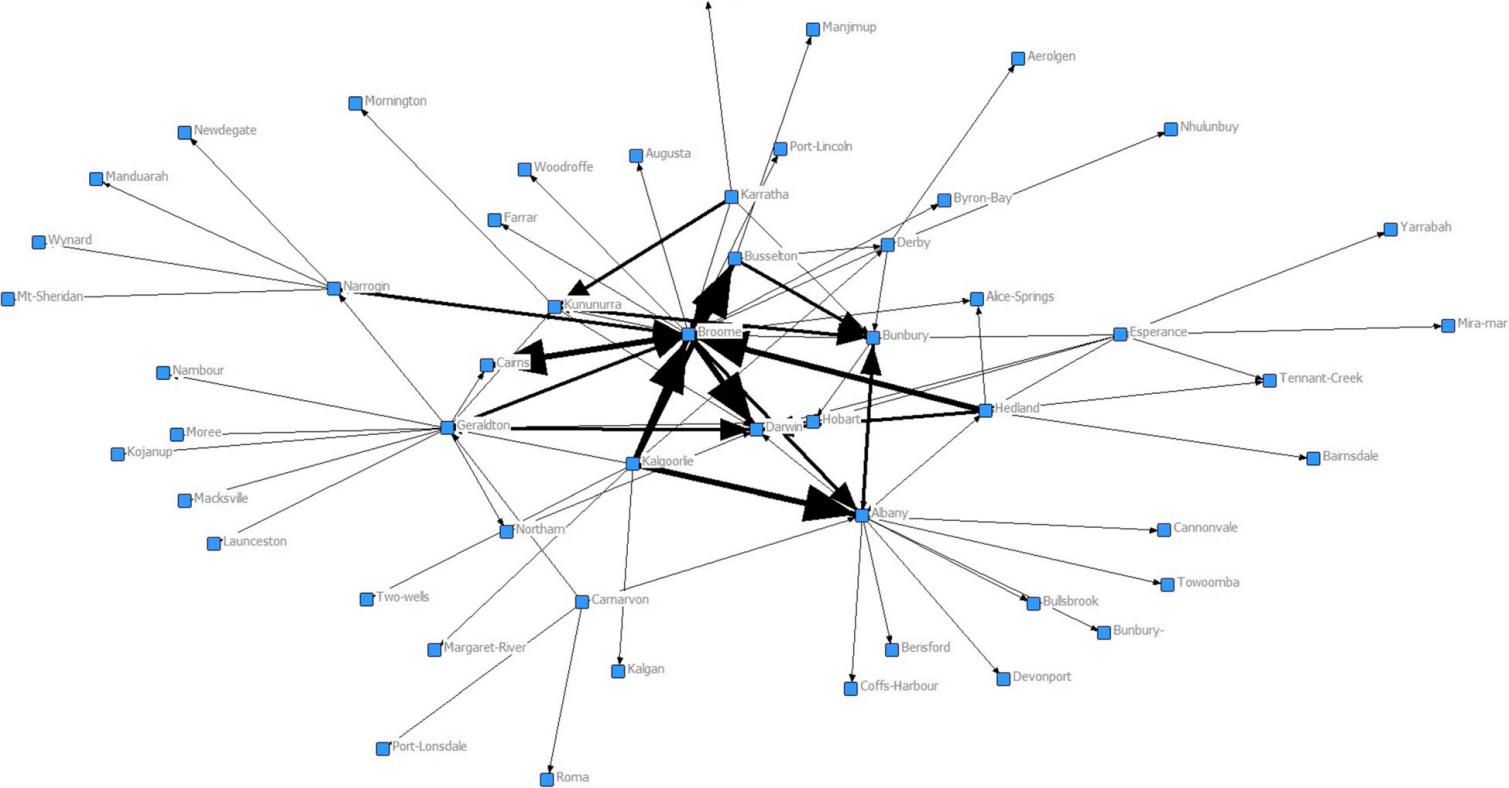
Social Network of Rural Clinical School Western Australia Edge Array showing density of connections from originating sites to work locations

Figure 2 also shows that the focus of all the directional ties for rural-working alumni was on the remote town of Broome, which the UCInet Netdraw programme calculated as being at the centre of the directional graph. That is, Broome was the town most connected (degree) and also most equidistant to all other town relationships (betweenness) expressed by the graduates. The strength of these relationships are numerically expressed in Table 1, which shows that Broome had the highest degree and betweenness scores calculated by UCInet, based on Broome having the highest number of both incoming (21) and outgoing (23) ties. These data are even more clearly demonstrated in Fig. 3, with weighted (valued) lines showing the aggregated connections between sites, with Broome again at the centre.

**Table 1:** 2017 UCINet / Netdraw analyses of tie characteristics for each Rural Clinical School of Western Australia node. (Town size data from the Australian Bureau of Statistics, SLA 2/Significant Urban Area level data, 2016 Census data, AHPRA 2017 workplace location data)

RCSWA SITE	Year site established	Total RCS placements (PGY2–15) *N* = 706	Number in rural Australia. (% total)	Number recruited to RCS site in 2017	Remoteness (ASGC-RA)	Remoteness (MMM)	Town population	Degree	Between-ness	Closeness
Broome	2002	84	28 (33)	26	RA4	6	13,984	18	570.769	144.000
Albany	2005	84	14 (16)	15	RA3	3	33,145	13	396.893	161.000
Geraldton	2002	96	17 (18)	5	RA3	3	37,432	14	378.462	159.000
Kalgoorlie	2002	117	17 (14.5)	0	RA3	3	29,873	8	198.000	165.000
Bunbury	2007	83	15 (18)	15	RA2	2	72,402	8	112.700	166.000
Esperance	2004	32	10 (31)	2	RA4	6	12,107	7	115.393	195.000
Port Hedland	2002	55	11 (20)	3	RA4	6	15,828	7	118.467	170.000
Kununurra	2011	12	4 (33)	4	RA4	6	7155	6	63.036	175.000
Narrogin	2007	27	7 (26)	3	RA3	5	4725	6	198.000	176.000
Derby	2005	27	6 (22)	3	RA5	7	7705	6	103.200	180.000
Busselton	2009	36	9 (25)	5	RA2	3	36,616	5	53.200	181.000
Carnarvon	2008	19	5 (26)	0	RA3	6	5160	4	113.000	186.000
Karratha	2006	30	5 (16)	0	RA4	6	15,828	4	51.000	186.000
Northam	2014	4	1 (25)	1	RA2	4	11,112	2	0.000	195.000

Examination of the direction of ties for graduates from Broome showed a focus on work in Northern Australia. As well as connections to northern sites in the state of Western Australia (Broome, Derby and Kununurra), graduates also connected with northern rural towns in the Northern Territory (Darwin, Alice Springs, Tennant Creek) and Queensland (Cairns). The town of Derby, 200 km north of Broome, also showed this same Northern preponderance with four of its five connections also being with northern sites around Australia (Broome, Derby, Nhulunbuy, Aeroglen).

Other sites were examples of potentially developing centrality: Albany received medical graduates from 11 sites, contributed graduates to 14 other sites, and had intermediate degree and betweenness scores.

Some towns with low degree scores because they received relatively few graduates nevertheless contributed workforce to other sites, as shown by their stronger “betweenness” scores: Kalgoorlie, and Narrogin only receiving a combined total of three doctors but sent out a combined total of 21 graduates to other rural / remote towns.

Since these data all relate to single “from” and “to” connections, closeness scores were similar for all sites.

**Fig. 4:**
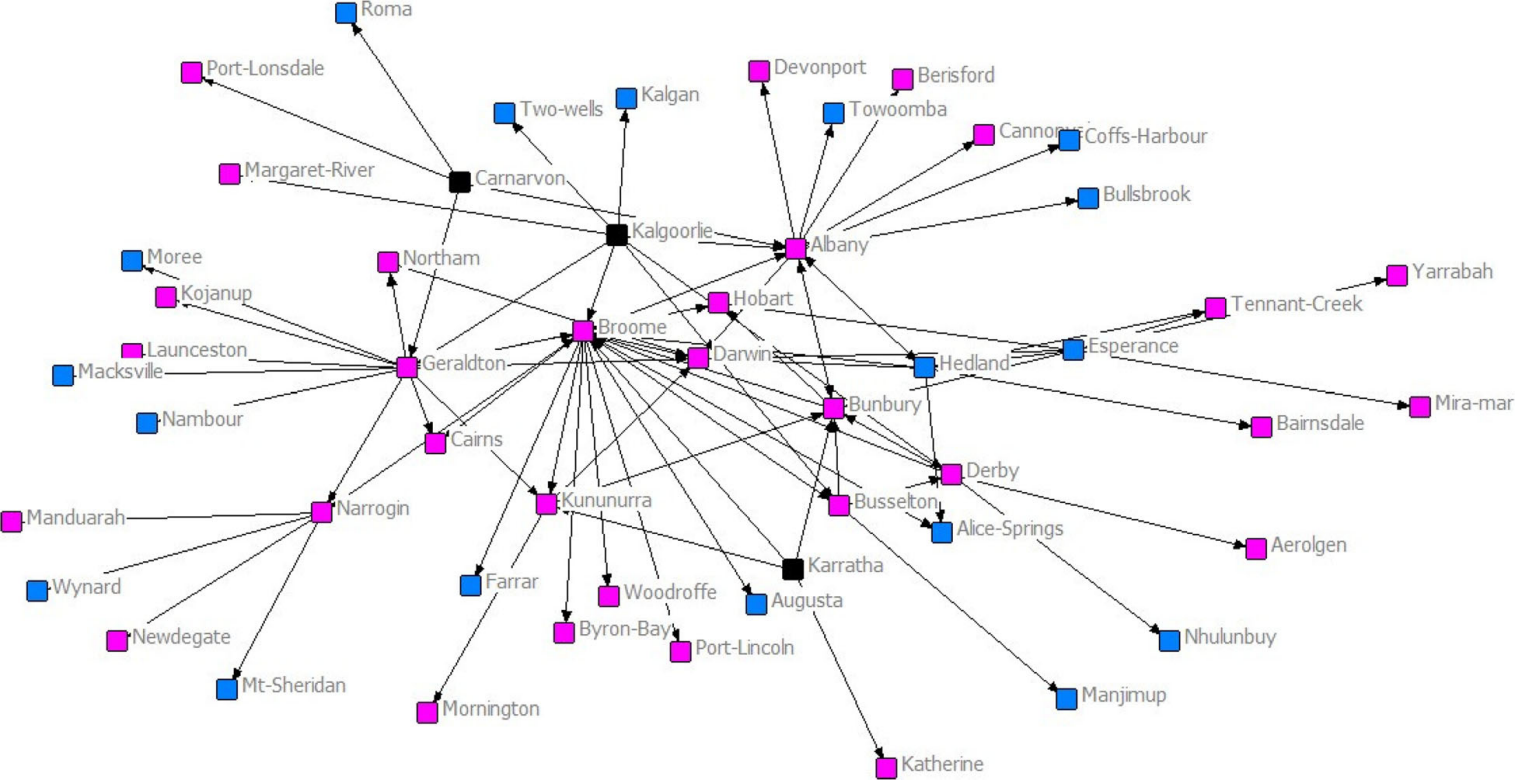
Social Network of Female (Pink) versus Male (Blue) rurally located graduates of RCSWA. Black nodes received no graduates in 2017

Examination of further attributes of the network indicated that a large number of women were attracted to rural towns around Australia (Fig. 4).

Reflexive ties – meaning doctors whose 2017 location was at the same site as their RCSWA training- cannot be shown graphically, but graduate return to the same site as their site of training were as follows: Broome and Bunbury both had five returning graduates, Albany and Esperance had two, the towns of Busselton and Derby had one. These data, using information given in Table 1, show that a spread of town sizes had same-site retention.

## Discussion

Reflecting trends worldwide, Western Australia is a strongly urbanised state, with approximately 80% of its population living in its one capital city [25]. The outcomes of this study show that the experience of a longitudinal integrated clerkship through RCSWA re-distributed 17.5% of its medical workforce, with a particular focus on the North of the state, and of the continent. This cannot simply be explained by graduates’ return to their own rural origins, since although 25% of RCSWA recruits are rural origin [9], very few in general come from the north [26], and in this cohort only one returned there. Instead it seems that the RCSWA, which brings pre-graduate students to longitudinal immersion in Western Australian rural towns, disperses them rurally in substantial numbers, and that it does so in a clearly geographically patterned way. We argue here that there is something in the graduates’ experience of longitudinal integrated clerkship that re-orients them to rural work in general and attracts them north in particular.

The concentration of new medical workforce in the remote north of Western Australia support the notion that RCSWA can be considered a form of “bridging capital” in social capital theory [27], or a weak tie in Social Network Analysis [28]. That is, the School brings students to Western Australian towns with which they had little long term prior contact [26] and this new relationship was sufficient to re-orient graduates’ sense of what postgraduate opportunities are available to them. Social capital is clearly focused in Northern regions of Western Australia, with one node – Broome - being its mainstay, as demonstrated by Social Network Analysis measures, particularly with respect to its substantial “betweenness” score which is indicative of its centrality [24]. This was a potentially unexpected result, because Broome is both smaller and more remote than larger and more accessible regional towns that could be expected to be attractive for new graduates [20].

In its favour, Broome is a tourist town for half the year when the weather is more clement, and to some extent this may contribute to attractiveness. However prior to the commencement of RCSWA, Broome had very limited medical training occurring, and had challenges recruiting GPs [29].

There are a number of features that may contribute to Broome’s attractive effect [20]. However significant to the present discussion, Broome includes many of the SNA characteristics of being a “bridge, broker and boundary spanner”, as summarized by Long et al. (2013), which have not previously been discussed. Long et al. (2013) state that “collaborative networks by definition, seek to bring disparate groups together so that they can work effectively and synergistically together. Brokers can support the controlled transfer of specialised knowledge between groups, increase cooperation by liaising with people from both sides of the gap, and improve efficiency by introducing “good ideas” from one isolated setting into another.” (p158) This precisely describes the role of Broome in the remote Northwest: the RCSWA has established this site as a research centre, with multiple grants from National funders including programme grants from the Australian Government’s National Health and Medical Research Council; its particular emphasis is on translational research that improves health outcomes for Aboriginal people; it is the principle support location for Aboriginal Medical Services in the Kimberley through the Kimberley Aboriginal Medical Service, with which RCSWA is in partnership for undergraduate and postgraduate medical education, Kimberley medical guideline development, and research. Through these various activities, Broome acts as a broker for rural and remote health, translating specialized knowledge into service provision guidelines, and introducing innovation into practice. Interestingly, these multiple activities also represent the creation of a teaching-research hub which has been advocated as central to workforce development [30].

The strong teaching and research advocacy of the lead RCSWA clinical, teaching and research academic, who also set up the Broome node, may play an additional important part in the relational aspects of this hub. Graduates who chose to locate in Broome anecdotally attributed their site attraction to the mentorship offered by this individual and by senior staff of the local hospital. These data suggest the significant workforce benefits that may result from mentorship within a remote medical teaching-research nexus.

It is interesting in this regard to contrast Broome’s result with other sites of similar – or larger size. For example, the coastal sites of Geraldton and Karratha, or the inland site of Kalgoorlie, also have substantial Aboriginal populations, and so offer opportunities to be socially accountable. Their lack of attractive effect suggests that Broome’s appeal has a more complex basis than social need.

Existing data on the creation of rural workforce also suggests that geography significantly affects the attractiveness of rural work. Coastal towns, for example, are more attractive than inland towns [20]. However as already observed for this study, even large coastal towns such as Geraldton and Bunbury, with significant amenities, may be less attractive new graduates than small towns with a large social capital. The present data are consistent with the strong attractive status of similarly remote regions in the remote north of Canada which also have a newly established university/research presence [31].

The other noteworthy result shown by SNA was for female graduates of RCSWA, who provided more than 60% of the rural graduate workforce distributed across the country. Such recruitment is in marked contrast to the traditional male domination of rural medical workforce [32]. It suggests that, where women are recruited in large numbers [9], the social capital inherent in the RCSWA project is able to attract and retain a proportionate number of females in subsequent work. Previous work suggests that social isolation is one of the primary negative aspects of rural practice for women [33]. This study’s positive result suggests that RCSWA graduates may be in the process of setting up new positive rural social networks for recently graduated women. It is likely to be relevant that the School as a whole has also recruited substantial numbers of experienced female doctors as teachers, which also runs counter to other rural workforce statistics [34], and further suggests that the social connectivity of RCSWAs may be positive to women in long term rural practice.

The new geographical relationships described here may be considered a form of “relational capital” [35] whereby new graduates both contribute to and receive from relationships in a given node in a way that has not previously described in the medical workforce literature. We assume that the results we report are the consequence of new relationships that developed in the year spent rurally, because the towns represented here have relatively small contributions of medical students to Western Australian medical programmes overall, and so supply little in the way of a priori relationships for alumni [26]. The way that graduates returned to sites other than their original RCSWA site further suggests that the School as a whole is acting as a “bridge, broker and boundary spanner” to rural work in a way that social network analysis describes [36].

This premise is given credence by the way that pre-existing connections between the North of Western Australia (Broome, Kununurra and Derby) and the Northern Territory (Darwin, Tennant Creek and Alice Springs) are also reflected in our medical graduate’s ties. The pre-existing relationship between towns in the “top end” of Australia comprise a coherent network in the tropical North [37], and includes strong connections between Northern Aboriginal peoples. The fact that graduate’s movements in this study reflected a similar network pattern – including northern towns outside of Western Australia which are not involved in teaching for the RCSWA year - suggests that the graduate ties we report are socially responsive.

### Limitations and future directions

This study did not intend to examine the impact of RCSWA on workforce composition. The study does not attempt to follow graduate locations over time, nor to compare the rural graduates with either urban background graduates or others who did not experience RCSWA. Such important data are being collected and reported in a separate and ongoing set of studies [9, 11, 21, 38], which have clearly shown RCSWA’s workforce impacts. For the same reason, we have not sought the individual characteristics of graduates who have moved north, but rather to show that there is, in fact, a northern trend. However, to extend the present study, it would be interesting to find out directly from graduates the reason/s why they went to different locations.

## Conclusions

Not only do these data show new patterns of workplace relationships for medical graduates, they also highlight some unexpected results. For example the largest regional town in Western Australia (Bunbury) did not emerge as a strong attractor in Social Network Analysis. This suggests that factors in addition to size, economic prosperity and coastal location [20] are important to recruitment.

In other words, recruitment may be sensitive to non-pragmatic factors such as social networks and their social capital. To this end, the data presented here using Social Network Analysis show that the RCSWA, which has been offering rural longitudinal integrated clerkships for sixteen years, is developing a new set of workforce based ties for its graduates that have not previously been described. These data demonstrate the extent to which social networks, as described by SNA, are involved in growing the rural workforce in regional Australia. These data are additionally relevant to universities strategically setting up undergraduate rural placement programmes, as they suggest aspects of the social milieu that are likely to be associated with subsequent workforce outcomes.

## Abbreviations

AHPRA: Australian Health Practitioner Regulation Agency
PGY: PostGraduate Year
RA: Remoteness Area
RCS: Rural Clinical School
RCSWA: Rural Clinical School of Western Australia
SNA: Social Network Analysis
